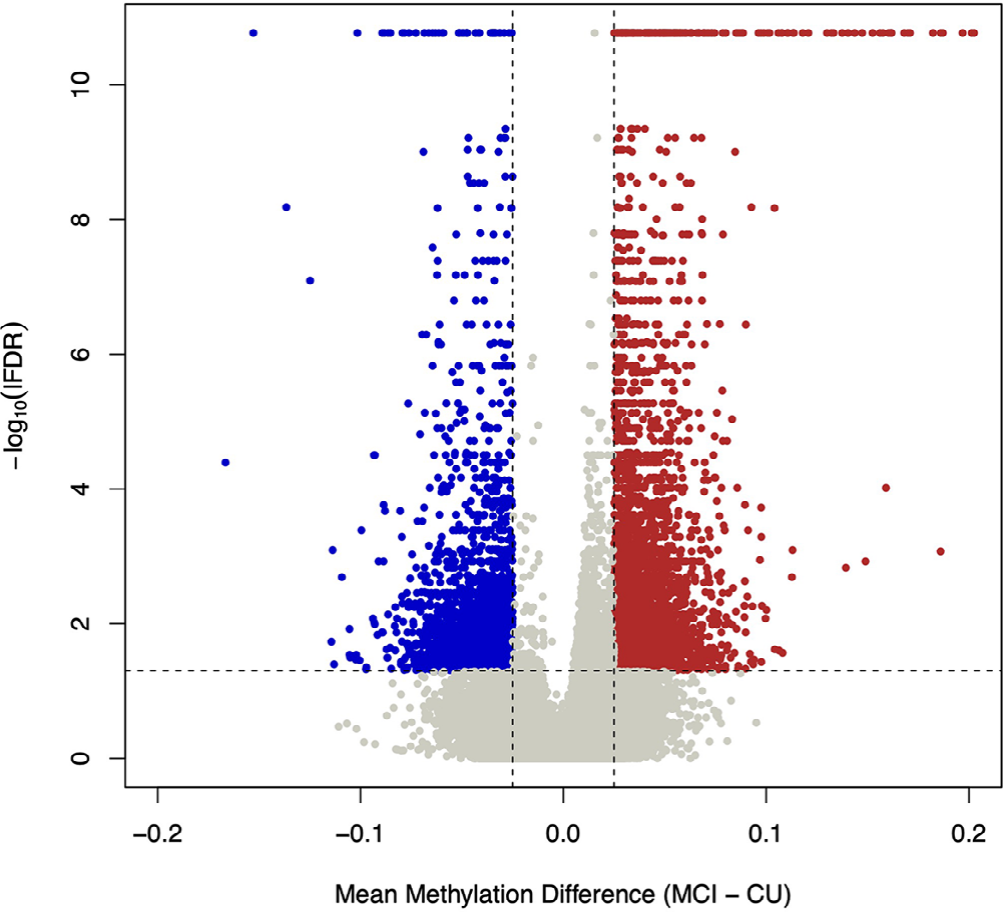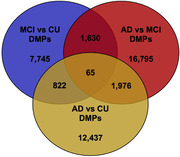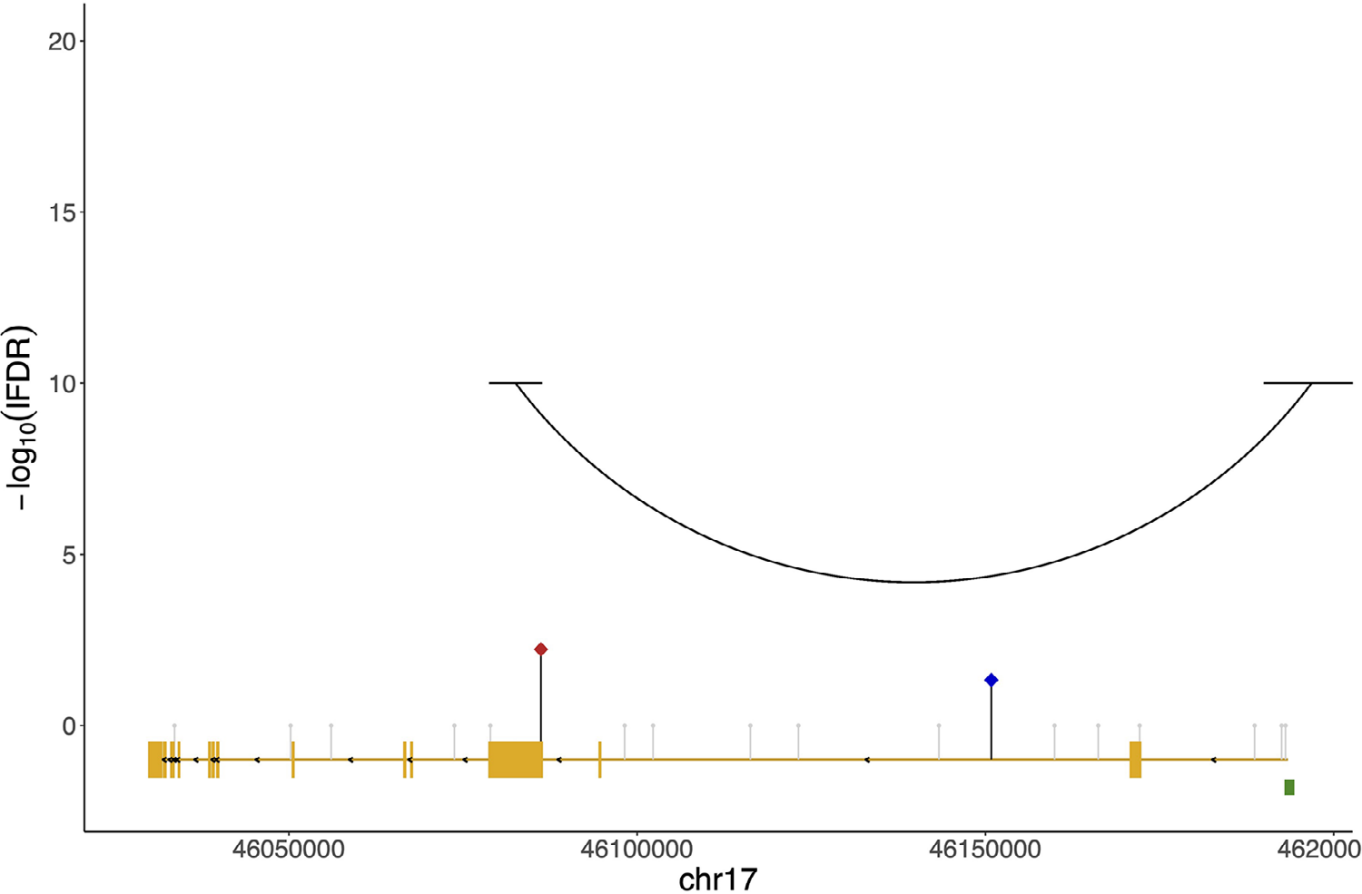# Whole genome methylation sequencing in blood identifies extensive differential DNA methylation in mild cognitive impairment (MCI)

**DOI:** 10.1002/alz.088557

**Published:** 2025-01-09

**Authors:** Andy Madrid, Coleman E Breen, Ligia A Papale, Lindsay R. Clark, Sanjay Asthana, Sterling C. Johnson, Sündüz Keles, Kirk J. Hogan, Reid S Alisch

**Affiliations:** ^1^ University of Wisconsin School of Medicine and Public Health, Madison, WI USA; ^2^ University of Wisconsin‐Madison, Madison, WI USA; ^3^ University of Wisconsin‐Madison School of Medicine and Public Health, Madison, WI USA; ^4^ University of Wisconsin ‐ Madison, Madison, WI USA

## Abstract

**Background:**

Whole genome methylation sequencing (WGMS) in blood identifies extensive differential DNA methylation between persons who are cognitively unimpaired (CU) and those with late‐onset dementia due to Alzheimer’s disease (AD). Here we investigate differentially methylated positions (DMPs) in persons with mild cognitive impairment (MCI) compared to persons with and without AD.

**Method:**

WGMS data quantified DNA methylation levels at 25,406,945 CpG loci in 382 blood samples from 99 persons with MCI, 109 persons with AD and 174 cognitively unimpaired persons in the Wisconsin Alzheimer’s Disease Research Center (WADRC) and the Wisconsin Registry for Alzheimer’s Prevention (WRAP). Beta‐binomial models were developed to test for significant DNA methylation levels between groups, accounting for batch effects, estimated white blood cell proportions, sex, body mass index (BMI), and age. P‐values were corrected and a local false discovery rate (lFDR) was computed.

**Result:**

WGMS identified 10,262 MCI‐associated DMPs (Figure 1). We identified 1,984 genes comprising one or more DMPs (*e.g*., *ACE*, *ANK3*, and *HLA‐DQA1*) encode proteins that participate in biological pathways relevant to neuronal function including synapse organization, dendrite formation and potassium ion transmembrane transport. Interrogation of DMPs among all two‐group comparisons (*i.e.*, MCI:CU, AD:CU, and AD:MCI) identified 65 overlapping DMPs (Figure 2). Twelve of the overlapping DMPs are associated with 4 genes. Neighboring *TDG* and *C12orf73* share 10 DMPs on chromosome 12q23.3, *FHIT* and *RXFP1* comprise 1 DMP each. The remaining shared 53 DMPs are intergenic. We further investigated DMPs with progressive changes in DNA methylation levels from CU to MCI to AD. We identified 570 DMPs with increasing or decreasing DNA methylation levels were identified, including DMPs in enhancer‐promoter interactions in genes previously associated with AD, including *KANSL1* (Figure 3).

**Conclusion:**

WGMS identifies differentially methylated CpGs in known and newly detected genes and enhancers in blood from persons with and without MCI and AD and reveals progressive dysregulation of DNA methylation levels between CU, MCI, and AD.